# SIRT2‐mediated deacetylation of ACLY promotes the progression of oesophageal squamous cell carcinoma

**DOI:** 10.1111/jcmm.18129

**Published:** 2024-03-01

**Authors:** Xueying Zhang, Yue Xu, Shenglei Li, Yue Qin, Guangzhao Zhu, Qing Zhang, Yanting Zhang, Fangxia Guan, Tianli Fan, Hongtao Liu

**Affiliations:** ^1^ School of Life Sciences Zhengzhou University Zhengzhou Henan China; ^2^ Department of Pathology The First Affiliated Hospital of Zhengzhou University Zhengzhou Henan China; ^3^ Translational Medicine Research Center Zhengzhou People's Hospital Zhengzhou Henan China; ^4^ Department of Pharmacology, School of Basic Medicine Zhengzhou University Zhengzhou Henan China

**Keywords:** acetylation, ATP citrate lyase, BMS‐303141, lipid metabolism, oesophageal squamous cell carcinoma, sirtuin 2

## Abstract

ATP citrate lyase (ACLY), as a key enzyme in lipid metabolism, plays an important role in energy metabolism and lipid biosynthesis of a variety of tumours. Many studies have shown that ACLY is highly expressed in various tumours, and its pharmacological or gene inhibition significantly inhibits tumour growth and progression. However, the roles of ACLY in oesophageal squamous cell carcinoma (ESCC) remain unclear. Here, our data showed that ACLY inhibitor significantly attenuated cell proliferation, migration, invasion and lipid synthesis in different ESCC cell lines, whereas the proliferation, migration, invasion and lipid synthesis of ESCC cells were enhanced after ACLY overexpression. Furthermore, ACLY inhibitor dramatically suppressed tumour growth and lipid metabolism in ESCC cells xenografted tumour model, whereas ACLY overexpression displayed the opposite effect. Mechanistically, ACLY protein harboured acetylated modification and interacted with SIRT2 protein in ESCC cells. The SIRT2 inhibitor AGK2 significantly increased the acetylation level of ACLY protein and inhibited the proliferation and migration of ESCC cells, while overexpression of ACLY partially reversed the inhibitory effect of AGK2 on ESCC cells. Overall, these results suggest that targeting the SIRT2/ACLY signalling axis may be a potential therapeutic strategy for ESCC patients.

## 1 INTRODUCTION

Oesophageal cancer (ESCA) is a malignant disease that greatly threatens human health, its incidence and mortality rank seventh and sixth among all cancers, respectively, and the 5‐year survival rate of patients remains relatively poor.^1^, ^2^, ^3^, ^4^ ESCA includes two major histological subtypes: oesophageal squamous cell carcinoma (ESCC) and oesophageal adenocarcinoma (EAC), of which approximately 87% of ESCA are ESCC, and 11% are EAC worldwide.^5^, ^6^, ^7^, ^8^, ^9^ Furthermore, the pathogenesis and aetiology of ESCC are poorly understood, and it is often diagnosed with extremely aggressive clinical features or at advanced stage.^10^ At present, the treatment strategies for ESCC patients are radiotherapy and chemotherapy, which often evoke strong toxic and side effects. To improve the treatment outcomes of patients with advanced ESCC, chemotherapy combined with immunotherapy and molecular‐targeted therapy have obtained widespread attention in a clinic.^11^ Therefore, to better improve the therapeutic effect of ESCC patients, it is urgent to seek suitable therapeutic targets and low‐toxic drugs.

Metabolic reprogramming is one of the typical biological characteristics of tumours, and the most well‐known metabolic reprogramming includes glucose metabolism^12^ and glutamine metabolism.^13^ Recently, the reprogramming of lipid metabolism has become the symbol of a new generation of cancer and may be the potential therapeutic target for many different types of cancer.^14^ Many studies have shown that tumours need to resynthesize fatty acids to meet their growth and proliferation needs, which is mainly due to the fact that lipids are not only components of biological membranes but also are implicated in the regulation of signal transduction.^15^ Increasing evidence has demonstrated that blocking some key enzymes in the fatty acid synthesis pathway can reduce the uptake of lipids by tumours, thereby exerting anti‐tumour effects, such as ATP citrate lyase (ACLY), Acetyl‐CoA synthetase (ACS), Acetyl‐CoA carboxylase 1 (ACC1), Fatty acid synthase (FASN), Sterol regulatory element binding transcription factor 1 (SREBF1), etc.^16^, ^17^, ^18^, ^19^ ACLY catalyses the Mg‐ATP‐dependent conversion of citrate and CoA to acetyl‐CoA and oxaloacetate.^20^, ^21^ Acetyl‐CoA supports protein acetylation and de novo synthesis of lipids, while oxaloacetate maintains nucleotide and polyamine synthesis and the regeneration of NADPH and H^+^. ACLY is a central enzyme‐linking glycolysis and lipid metabolism, decomposes cytosolic citrate to promote glycolytic activity and de novo lipid synthesis by producing acetyl‐CoA.^22^ Many recent studies have shown that ACLY is highly expressed in multiple different types of tumours, including glioblastoma,^23^ castration‐resistant prostate cancer,^24^ cervical cancer,^25^ lung cancer^25^, ^26^, ^27^ and colon cancer.^28^ ACLY is closely related to local tumour stage and overall survival in patients with non‐small‐cell lung cancer,^29^ and lung cancer patients with high ACLY expression have a poorer overall survival rate.^30^ In vitro studies have shown that inhibition of ACLY can limit tumour proliferation and induce apoptosis.^31^ These studies suggest that ACLY plays an important role in the progression of various types of malignant tumours. However, the roles and molecular mechanisms of ACLY in the development and progression of ESCC remain still unknown.

Here, we investigated the expression patterns of ACLY in ESCC tissues and cells and explored the roles of ACLY in cell proliferation, migration, invasion and lipid metabolism in ESCC cells. Subsequently, we further dissected the effects of ACLY on tumour growth and lipid metabolism through animal experiments. Finally, we investigated the tumour‐promoting effect and molecular mechanism of Sirtuin 2 (SIRT2)‐mediated ACLY deacetylation in ESCC. Overall, the current study provides new experimental evidence for targeted therapy of ESCC patients by targeting the SIRT2/ACLY signalling axis.

## 2 MATERIALS AND METHODS

### 2.1 Bioinformatics assay

ACLY expression in pan‐cancer was determined by the TCGA database from Sangerbox (http://vip.sangerbox.com/home.html). The expressions of ACLY were detected using the TCGA database integrated into GEPIA (http://gepia.cancer‐pku.cn/), UALCAN (http://ualcan.path.uab.edu/) and Starbase (https://starbase.sysu.edu.cn/).

### 2.2 ESCC samples, cell lines and cell culture

Thirty‐one cases of ESCC samples and paired normal samples were obtained from the First Affiliated Hospital of Zhengzhou University, which was approved by the Research and Ethics Committee of Zhengzhou University. Human ESCC cell lines including Eca109 and KYSE450 and normal oesophageal epithelial cell Het‐1A were purchased from Qingqi Biotechnology Development Co., Ltd (Shanghai, China), and ESCC cell KYSE150 was purchased from Procell Life Science & Technology Co., Ltd (Wuhan, China). The above cell lines were cultured in RPMI 1640 medium supplemented with 10% FBS and double antibiotics including Penicillin and Streptomycin. These cells were placed in an incubator with 5% CO_2_ at 37°C.

### 2.3 Transfection of plasmid

ESCC cells were inoculated into six‐well plates according to 2 × 10^5^ cells per well and were cultured in a CO_2_ incubator for 24 h. When cell confluence reached about 80%, 1.5 μg empty vector pCMV‐Myc (NC) and 1.5 μg ACLY overexpression vector pCMV‐Myc‐ACLY (ACLY OE) were transfected to ESCC cells by Lipo8000™ Transfection Reagent (Beyotime Biotechnology, Shanghai, China) according to manufacturer's protocol.

### 2.4 Cell counting Kit‐8 (CCK‐8) assay

Cell proliferation was determined using a CCK‐8 kit (Dojindo Laboratories, Japan) according to the manufacturer's instruction. Briefly, ESCC cells were inoculated into 96‐well plates with 3 × 10^3^ cells per well, cultured for 24 h and treated with BMS‐303141 (Abmole, USA) at different concentrations (0, 10, 20, 30, 40, 50, 60, 70 and 80 μM), or SIRT2 inhibitor AGK2 (MCE, USA) at different concentrations (0, 4, 8, 12, 16 and 20 μM). Ten per cent of CCK‐8 was added to the 96‐well plates according to the manufacturer's instructions at indicated time points including 24, 48, 72 and 96 h. The absorbance value at 450 nm was measured 2 h after the addition of CCK‐8 using a microplate reader. Similarly, the transfected ESCC cells were collected and inoculated into 96‐well plates at 3 × 10^3^ cells per well and replaced with an equal volume of 10% CCK‐8 medium at 12, 24, 36, 48, 60 and 72 h, respectively, and the absorbance value at 450 nm was detected 2 h after addition of CCK‐8 using a microplate reader.

### 2.5 EdU staining assay

Cell proliferation rate was determined using an EdU kit (RIBOBIO, Guangzhou, China) according to manufacturer's instruction. In brief, ESCC cells were inoculated into 48‐well plates with 1 × 10^4^ cells per well and incubated for 24 h. Subsequently, ESCC cells were collected 48 h after treatment with BMS‐303141 at various concentrations (0, 30, 50 and 70 μM) or AGK2 (20 μM) and transfection with NC and ACLY OE plasmids. Then, the treated cells were labelled with EdU reagent (RIBOBIO, Guangzhou, China) at a final concentration of 50 μM for 2 h, then washed with PBS buffer for 5 min, fixed with 4% paraformaldehyde for 30 min, and 200 μL of glycine (2 mg/mL) was added to the cells for incubation on a shaker for 5 min. Subsequently, destaining was performed using TritonX‐100 in a volume of 100 μL for 10 min. Followed by Apollo staining, the Apollo staining working solution was prepared according to manufacturer's instructions, and 100 μL of staining solution was added to each well and incubated for 30 min. It was then destained with PBS buffer containing 0.5% TritonX‐100 for 10 min. Finally, DNA staining was performed using 1× Hoechst33342. After staining, the excess dye was removed with PBS buffer, and pictures were taken with a fluorescence microscope.

### 2.6 Colony formation assay

ESCC cells treated with BMS‐303141 at various concentrations (0, 10, 20 and 30 μM) and transfected with NC plasmid and ACLY OE plasmid were collected and inoculated into 6‐well plates with 1 × 10^3^ cells per well, respectively. Then, ESCC cells were cultured for continuous 7–10 days until single‐cell colonies appeared, during which fresh medium was replaced. Finally, ESCC cells were fixed with 4% paraformaldehyde for 30 min and then stained with 0.1% crystal violet for 30 min.

### 2.7 Transwell assay

Cell migration and invasion were investigated using Transwell (Corning, USA), Briefly, the concentration of BMS‐303141 is 0, 30, 50, 70 μM, and the concentration of AGK2 is 0, 20 μM, respectively. Different treatment of ESCC cells was collected and cell density was adjusted to 5 × 10^4^/mL, which (200 μL per well) was inoculated into the upper chamber with uncoated (cell migration assay) or matrigel coated (cell invasion assay), and the lower chamber was filled with 600 μL culture medium containing 20% FBS. Then, cells were maintained in a 37°C 5% CO_2_ incubator for 48 h. Migratory or invasive cells were fixed with 4% paraformaldehyde for 30 min, stained with 0.1% crystal violet for 30 min and washed with PBS to reduce background. Images were captured using a microscope.

### 2.8 Nile red staining

The lipid content of ESCC cells was determined using Nile red staining (Solarbio, Beijing, China). Briefly, the treated ESCC Cells inoculated into 48‐well plates were fixed with 4% paraformaldehyde for 30 min. Then, cells were stained with 5 μg/mL Nile red solution for 30 min and then stained with DAPI working solution for 15 min. Finally, the excess dye was removed by rinsing with PBS, and the images were observed and obtained under a fluorescence microscope.

### 2.9 Quantitative real‐time PCR (qRT‐PCR)

Total RNA was isolated using a TRIzol kit (Solarbio, Beijing, China) and immediately transcribed into cDNA using a Prime‐Script RT kit (Takara, Japan). After reverse transcription, cDNA was amplified using SYBR Green PCR Master Mix (Takara, Japan) using the following primers: ACLY‐F: 5′‐TGAGGAAGCATCCGGAGGTA‐3′, ACLY‐R: 5′‐TCCGATGATGGTCACTCCCT‐3′, β‐actin‐F: 5′‐GCTCCTCCTGAGCGCAAG‐3′, β‐actin‐R: 5′‐CATCTGCTGGAAGGTGGACA‐3′. qRT‐PCR was performed on 384‐well plates using a real‐time PCR system. Each experiment was performed in triplicate with β‐actin as a control.

### 2.10 Western blot

To obtain total protein lysates, triturated tissue or cell pellets were lysed with cell lysate‐containing mixed protease inhibitors. The lysate was incubated on ice for 30 min and centrifuged at 12,000 *g* for 15 min at 4°C. The protein concentration of each sample was determined using the bicinchoninic acid (BCA) assay. Samples were loaded on 10% SDS‐PAGE. After electrophoresis, proteins in the gel were transferred to PVDF membranes. Membranes were blocked with 5% skimmed milk in TBST for 2 h at room temperature, then incubated with primary antibodies overnight at 4°C, followed by secondary antibodies for 2 h. The main antibodies include anti‐ACLY (Proteintech, Wuhan, China), anti‐SREBF1 (Proteintech, Wuhan, China), anti‐MOGAT2 (Proteintech, Wuhan, China), anti‐ACC1 (Proteintech, Wuhan, China), anti‐ACS (Proteintech, Wuhan, China), anti‐FASN (Proteintech, Wuhan, China), anti‐β‐actin (Proteintech, Wuhan, China), pan‐acetylated antibody (AC) (PTMBIO, Hangzhou, China), anti‐IgG (Proteintech, Wuhan, China), anti‐SIRT2 (Proteintech, Wuhan, China), anti‐HDAC1 (Proteintech, Wuhan, China) and anti‐PCAF (Proteintech, Wuhan, China). Immunoreactive bands were observed with an Azure C300 system.

### 2.11 Co‐immunoprecipitation (Co‐IP)

Co‐IP assay was performed to investigate the interaction protein using Co‐IP kit (Absin, Shanghai, China) according to manufacturer's instructions. Specifically, ESCC cells were fully lysed with 1 mL Co‐IP lysate at 4°C for 30 min, and then the total protein extracted was adjusted to the same concentration. In this study, 1–5 μg of ACLY or SIRT2 antibody was added to 500 μL protein mixture (containing 200–1000 μg of total protein), and cognate IgG was used as a control. The samples were rotated at 4°C overnight. Then, 5 μL protein A and 5 μL protein G were added to incubate at 4°C for 1 h, centrifuged at 12,000 *g* for 1 min, and the precipitate was washed three times using wash buffer. Then, the mixtures were centrifuged at 12,000 *g* for 1 min, and the precipitate was retained and resuspended with 20–40 μL of 1 × SDS sample buffer. Finally, the samples were heated at 100°C for 5 min, centrifuged at 14,000 *g* for 1 min, and the supernatant was used for Western blot assay.

### 2.12 Animal experiment

BALB/c nude mice aged 4–6 weeks were purchased from Beijing Vital River Laboratory Animal Technology Co., Ltd (Beijing, China). The animal experiment protocol was approved and conducted by the Institutional Committee for Use and Care of Laboratory Animals of Zhengzhou University. KYSE150 cells (2 × 10^6^ cells/mouse) were subcutaneously injected into the right side of the back of nude mice, and 5 mice were contained in each group. When the tumour volume reached about 100 mm^3^, nude mice were randomly divided into four groups: Control group and BMS‐303141 group, NC group and ACLY OE group. Control group and BMS‐303141 group were treated once a day by intraperitoneal injection with 100 μL per nude mouse. BMS‐303141 was first dissolved in DMSO as a 100 mg/mL stock solution. Control group: 40 μL PEG400 + 5 μL Tween 80 + saline 55 μL and 100 μL per nude mouse; BMS‐303141 group: 40 μL PEG400 + 5 μL Tween 80 + saline 54 μL+ 1 μL of BMS‐303141 stock solution. The treatment mode of NC group and ACLY OE group was intratumoral injection, and the treatment procedure was twice a week for continuous 2 weeks. The injection system of each NC group was 2.5 μg pCMV‐Myc empty plasmid + 5 μL in vivo DNA transfection reagent + 32.5 μL of 10% glucose solution; the injection system of each ACLY OE group was 2.5 μg of pCMV‐Myc‐ACLY plasmid + 5 μL of in vivo DNA transfection reagent + 32.5 μL of 10% glucose solution. From the day of treatment, the tumour volumes were measured every 2 days and the body weight of the mice was weighed and recorded. When the measurement was terminated, the nude mice were anaesthetised, photographed and recorded. The tumour tissue was peeled off from the subcutaneous tissue to detect the expression of lipid‐related proteins by Western blot.

### 2.13 Statistical analysis

All experimental data were statistically analysed using GraphPad Prism 8.0 software, and the data were expressed as the mean ± standard deviation of each group of replicates. The Student's *t* test was used to compare the means of two groups of samples that obeyed normal distribution and homogeneity of variance, otherwise, the Mann–Whitney *U* nonparametric test was used; three groups or more were compared using One‐way ANOVA analysis. A *p* less than 0.05 was considered to be a statistical difference.

## 3 RESULTS

### 3.1 ACLY is highly expressed in ESCA

**FIGURE 1:**
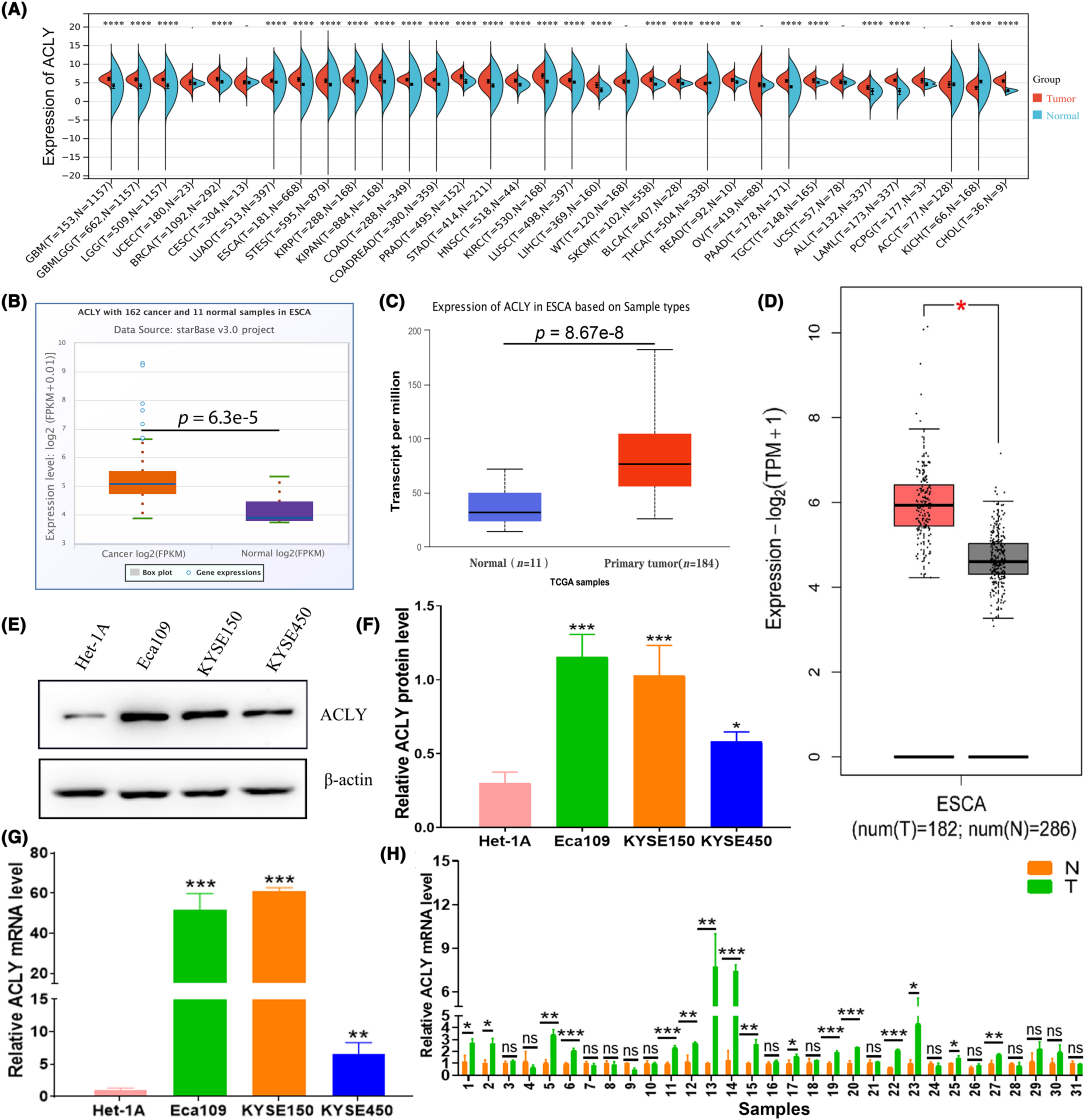
High expression of ATP citrate lyase (ACLY) in ESCA. (A) Differential expression assay of ACLY in pan‐cancer and normal tissues by Sangerbox; (B) StarBase v3.0 database investigation for ACLY expression in ESCA tissues; (C) UALCAN database assay for ACLY expression in ESCA tissues; (D) GEPIA database detection for ACLY expression in ESCA tissues; (E) Western blot assay for ACLY protein level in normal oesophageal epithelial cell Het‐1A and oesophageal squamous cell carcinoma (ESCC) cells including Eca109, KYSE150 and KYSE450; (F) Statistical assay for relative ACLY protein level in Het‐1A and ESCC cells; (G) qRT‐PCR assay for relative ACLY mRNA level in Het‐1A cells and ESCC cells; (H) qRT‐PCR assay for relative ACLY mRNA level in 31 ESCC tissues and paired normal tissues; *p < 0.05, **p < 0.01 and ***p < 0.001 represent statistical differences, ns, no significance.

We first investigated the expression of ACLY in pan‐cancer through the TCGA database Sangerbox. The results showed that ACLY was highly expressed in 25 of 34 different tumour types, including ESCA (Figure 1A). To further analyse the expression of ACLY in ESCA, three TCGA databases including StarBase v3.0, UALCAN and GEPIA were employed to unravel the expression of ACLY in ESCA tissues. The results demonstrated that the expression of ACLY in ESCA samples was significantly higher than that in normal samples (Figure 1B–D). Further investigation revealed that the protein and mRNA expression of ACLY in ESCC cells were extremely higher than those in Het‐1A cells (Figure 1E–G). Finally, we detected the expression of ACLY mRNA in 31 ESCC tissues and paired normal tissues by qRT‐PCR. The results showed that the expression of ACLY in 16 ESCC tissues was markedly higher than that in the corresponding normal tissues (Figure 1H). The above data suggest that ACLY displays high expression in ESCC tissues and cells.

### 3.2 ACLY inhibitor significantly suppresses the growth of ESCC in vitro and in vivo

**FIGURE 2:**
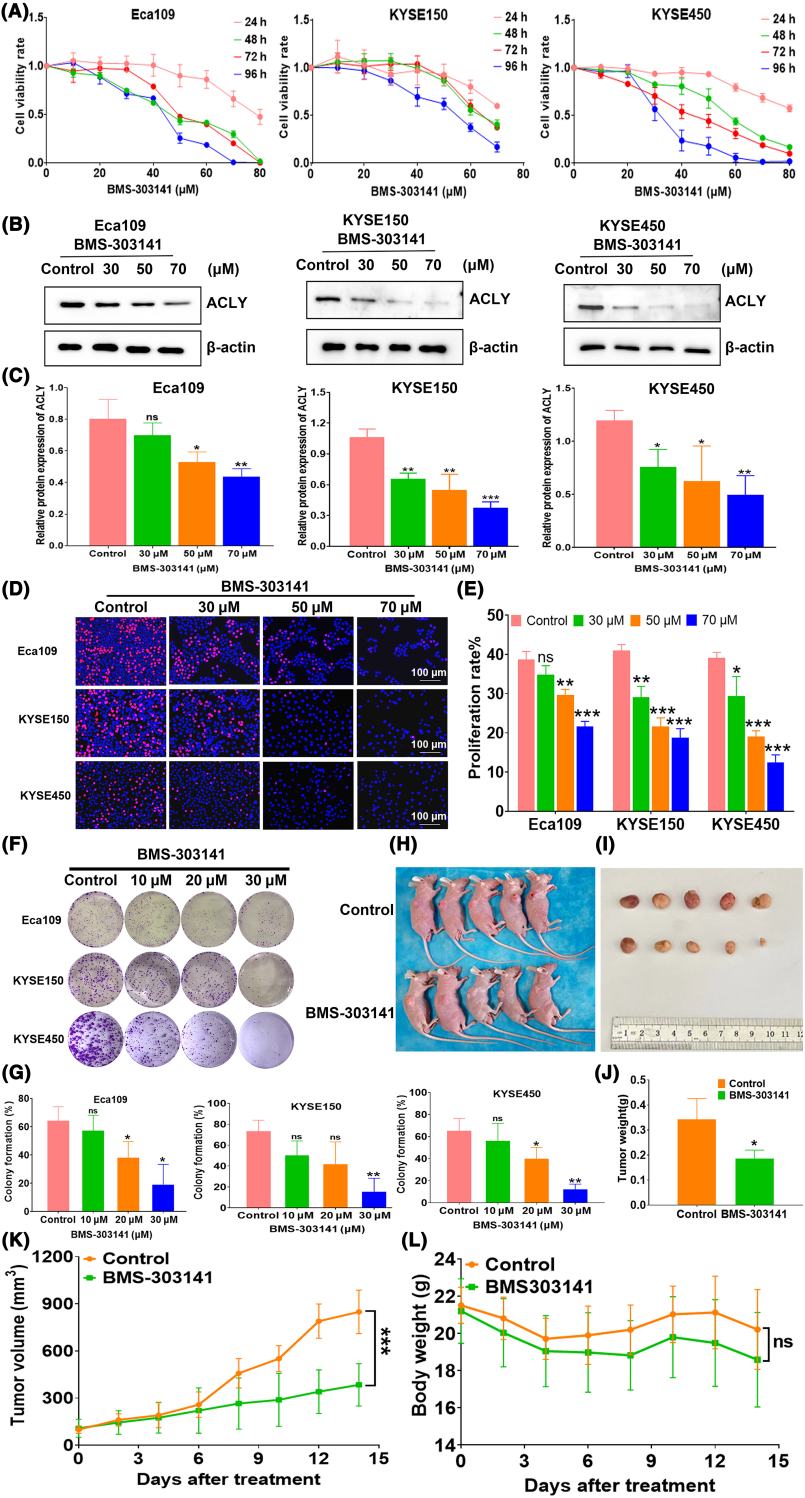
ATP citrate lyase (ACLY) inhibitor BMS‐303141 suppresses the growth of oesophageal squamous cell carcinoma (ESCC) in vitro and in vivo. (A) The inhibitory effects of BMS‐303141 on ESCC cell lines Eca109, KYSE150 and KYSE450 were investigated by CCK‐8 assay at 24, 48, 72 and 96 h after treatment with BMS‐303141 at different concentrations; (B) Western blot determination for ACLY protein level in Eca109, KYSE150 and KYSE450 cells treated with different concentrations of BMS‐303141; (C) Relative level of ACLY protein in Eca109, KYSE150 and KYSE450 cells treated with different concentrations of BMS‐303141; (D) EdU staining for determining cell proliferation rate of Eca109, KYSE150 and KYSE450 cells treated with different concentrations of BMS‐303141, blue: Hoechst‐stained nuclei; red: EdU‐stained proliferative cells; (E) Statistical assay for EdU‐positive cell numbers in Eca109, KYSE150 and KYSE450 cells treated with different concentrations of BMS‐303141; (F) The colony formation assay of Eca109, KYSE150 and KYSE450 cells treated with different concentrations of BMS‐303141; (G) Statistical assay for colony formation rate of Eca109, KYSE150 and KYSE450 cells treated with different concentrations of BMS‐303141; (H) Pictures of nude mice after BMS‐303141 treatment; (I) Pictures of the xenograft tumours after BMS‐303141 treatment; (J) Weight of xenograft tumours in nude mice; (K) Growth curves of xenograft tumours in nude mice in Control group and BMS‐303141 group; (L) Body weight curves of nude mice in Control group and BMS‐303141 group; *p < 0.05, **p < 0.01, ***p < 0.001, indicating statistical significance, ns: no significance.

To investigate the effects of ACLY inhibitor BMS‐303141 on ESCC proliferation, a CCK‐8 kit was used to detect the effect of BMS‐303141 on the survival rate of ESCC cells. The results showed that BMS‐303141 significantly inhibited the survival rate of ESCC cells in a time‐ and dose‐dependent manner (Figure 2A). Subsequently, the protein level of ACLY in Eca109, KYSE150 and KYSE450 treated with different concentrations of BMS‐303141 (0, 30, 50 and 70 μM) were determined by Western blot. The results demonstrated that the protein level of ACLY was significantly decreased after BMS‐303141 treatment (Figure 2B,C). The EdU staining revealed that the proportion of EdU‐positive cells in the three ESCC cell lines was dramatically reduced after BMS‐303141 treatment (Figure 2D,E). Besides, the results from colony formation demonstrated that BMS‐303141 at 30 μM significantly inhibited the colony formation ability of ESCC cells (Figure 2F,G). To explore the effect of the ACLY inhibitor BMS‐303141 on the growth of xenograft tumours in nude mice, we established subcutaneous tumour model using human ESCC cells KYSE150, and then BMS‐303141 was used to treat the tumour. We found that BMS‐303141 markedly suppressed tumour growth, compared with the control group (Figure 2H–K). In addition, there was no significant difference in the body weight of nude mice between the BMS‐303141 treatment group and the control group (Figure 2L). These results indicate that ACLY inhibitor significantly suppresses ESCC growth both in vitro and in vivo.

### 3.3 ACLY overexpression dramatically promotes the growth of ESCC in vitro and in vivo

**FIGURE 3:**
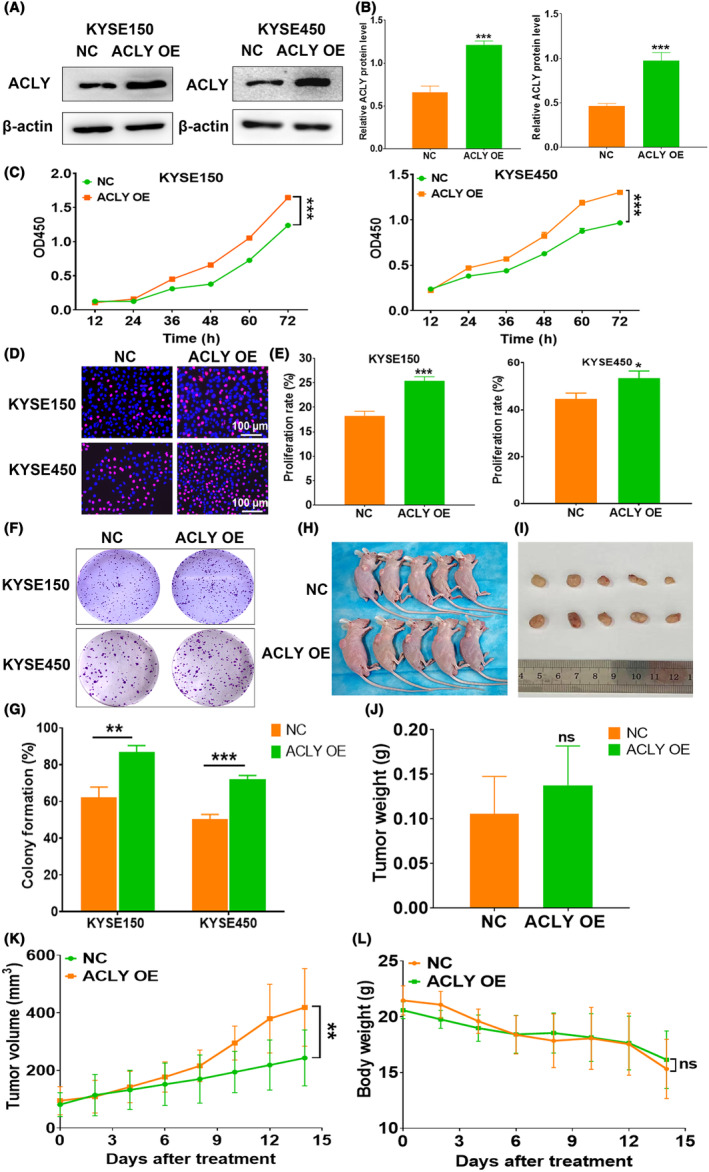
ATP citrate lyase (ACLY) overexpression promotes the growth of oesophageal squamous cell carcinoma (ESCC) in vitro and in vivo. (A) Western blot detection of ACLY protein expression level in KYSE150 and KYSE450 cells in NC and ACLY OE groups; (B) Relative ACLY protein level in NC and ACLY OE groups; (C) CCK‐8 detection of the proliferation ability of KYSE150 and KYSE450 cells in NC and ACLY OE groups; (D) EdU staining detection for the proliferation rate of KYSE150 and KYSE450 cells in NC and ACLY OE groups; (E) Statistical assay for EdU positive cell numbers in NC and ACLY OE groups; (F) Colony formation ability assay of KYSE150 and KYSE450 cells in NC and ACLY OE groups; (G) Statistical assay for colony formation rate in NC and ACLY OE groups; (H) Pictures of nude mice after treatment with NC and ACLY OE; (I) Pictures of the xenograft tumours after treatment with NC and ACLY OE; (J) Weight of xenograft tumours in nude mice after treatment with NC and ACLY OE; (K) Growth curves of xenograft tumours in nude mice in NC and ACLY OE groups; (L) Body weight curves of nude mice in NC and ACLY OE groups; *p < 0.05, **p < 0.01 and ***p < 0.001 indicate statistical differences, ns, no significance.

To further verify the role of ACLY overexpression in cell growth of ESCC cells, we transfected ACLY overexpression plasmid ACLY OE and NC into ESCC cells KYSE150 and KYSE450, respectively. Western blot was used to detect the expression of ACLY protein at 48 h. The results showed that the expression of ACLY protein in both KYSE150 and KYSE450 cells in the ACLY OE group was significantly higher than that in the NC group (Figure 3A,B). Further CCK‐8 experiment revealed that compared with the NC group, ACLY overexpression significantly promoted the proliferation of KYSE150 and KYSE450 cells (Figure 3C), which was also further validated by EdU staining (Figure 3D,E). In addition, the results of the colony formation experiments showed that overexpression of ACLY significantly promoted the colony formation of KYSE150 and KYSE450 cells (Figure 3F,G). These data indicated that ACLY overexpression significantly promoted the proliferation ability of ESCC cells in vitro. To further explore the role of ACLY overexpression in ESCC tumorigenesis by nude mice xenograft model, NC and ACLY OE plasmids were coated with liposomes and injected into each nude mouse by intratumoral injection. We found that ACLY overexpression promoted tumour growth in KYSE150 xenografted nude mice, and the tumour volume of ACLY OE group was larger than that of NC group (Figure 3H–K), but there was no significant difference in the body weight of nude mice (Figure 3L). These results indicate that ACLY overexpression significantly promotes the growth of ESCC cells in vitro and in vivo.

### 3.4 The effects of ACLY on migration and invasion of ESCC cells

**FIGURE 4:**
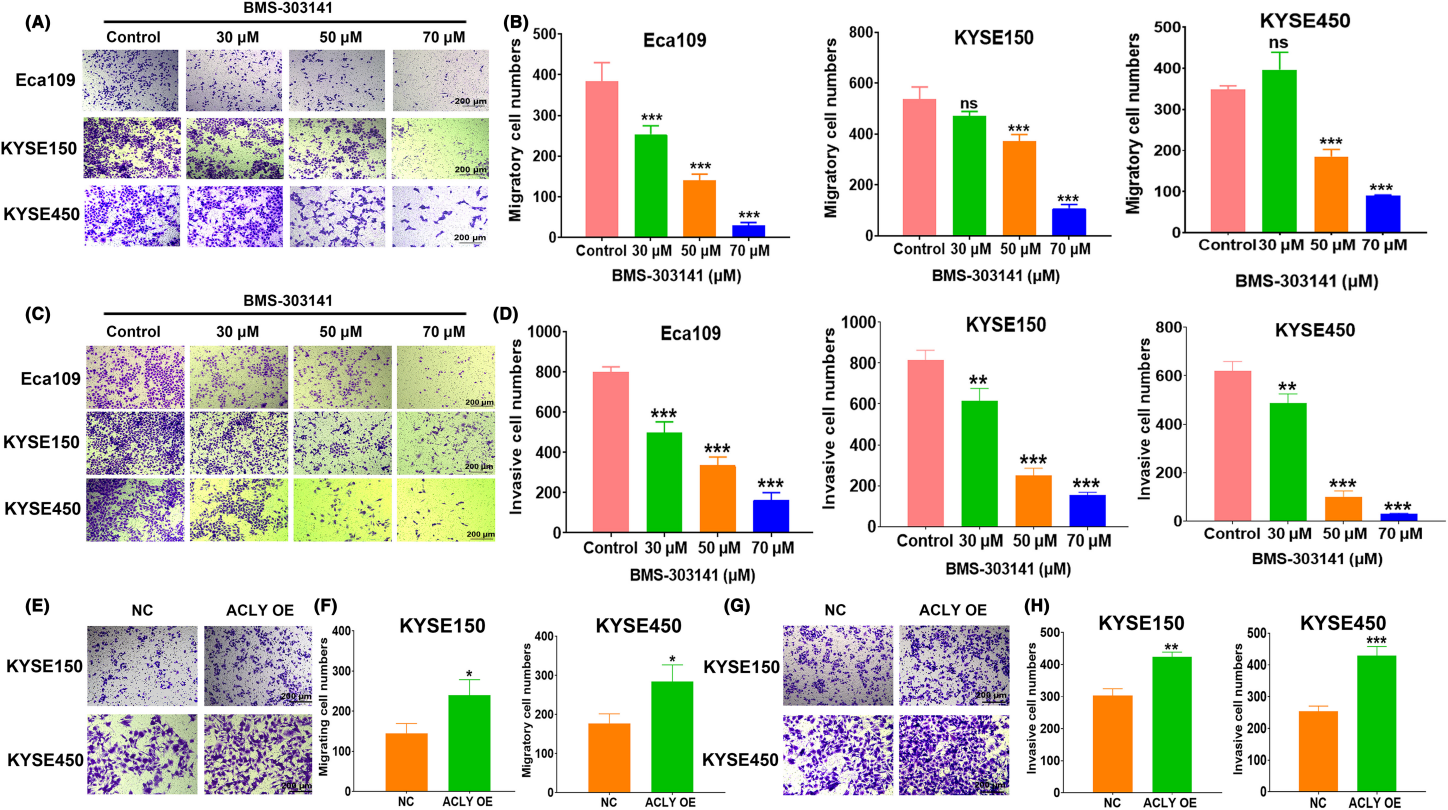
The effects of ATP citrate lyase (ACLY) on the migration and invasion of oesophageal squamous cell carcinoma (ESCC) cells. (A) Transwell experiment determination for migratory ability of Eca109, KYSE150 and KYSE450 after treatment with different concentrations of BMS‐303141; (B) Statistical assay for migratory cell numbers of Eca109, KYSE150 and KYSE450 cells after treatment with different concentrations of BMS‐303141; (C) Transwell experiment determination for invasive ability of ESCC cells Eca109, KYSE150 and KYSE450 after treatment with different concentrations of BMS‐303141; (D) Statistical assay for invasive cell numbers of Eca109, KYSE150 and KYSE450 cells after treatment with different concentrations of BMS‐303141; (E) Transwell experiment determination for migratory ability of KYSE150 and KYSE450 in NC and ACLY OE groups; (F) Statistical assay for migratory cell numbers of KYSE150 and KYSE450 cells in NC and ACLY OE groups; (G) Transwell experiment detection for invasive ability of KYSE150 and KYSE450 in NC and ACLY OE groups; (H) Statistical assay for invasive cell numbers of KYSE150 and KYSE450 cells in NC and ACLY OE groups; *p < 0.05, **p < 0.01 and ***p < 0.001 indicate a statistical difference, ns, no significance.

To detect the effect of ACLY inhibitors on the migration and invasion ability of ESCC cells, Transwell assay without or with Matrigel was performed after treatment with BMS‐303141 at 0, 30, 50 and 70 μM concentrations. The results showed that with the increase in BMS‐303141 concentration, the number of migratory (Figure 4A,B) and invasive cells (Figure 4C,D) gradually decreased in Eca109, KYSE150 and KYSE450. We further investigated the effect of ACLY overexpression on the migration and invasion ability of ESCC cells by the Transwell chamber. We found that the number of migratory cells (Figure 4E,F) and invasive cells (Figure 4G,H) in the ACLY OE group was significantly higher than that in the NC group. These results suggest that ACLY may play an important role in the regulation of migration and invasion of ESCC cells.

### 3.5 ACLY inhibitor significantly blocks lipid synthesis in ESCC cells in vitro and in vivo

**FIGURE 5:**
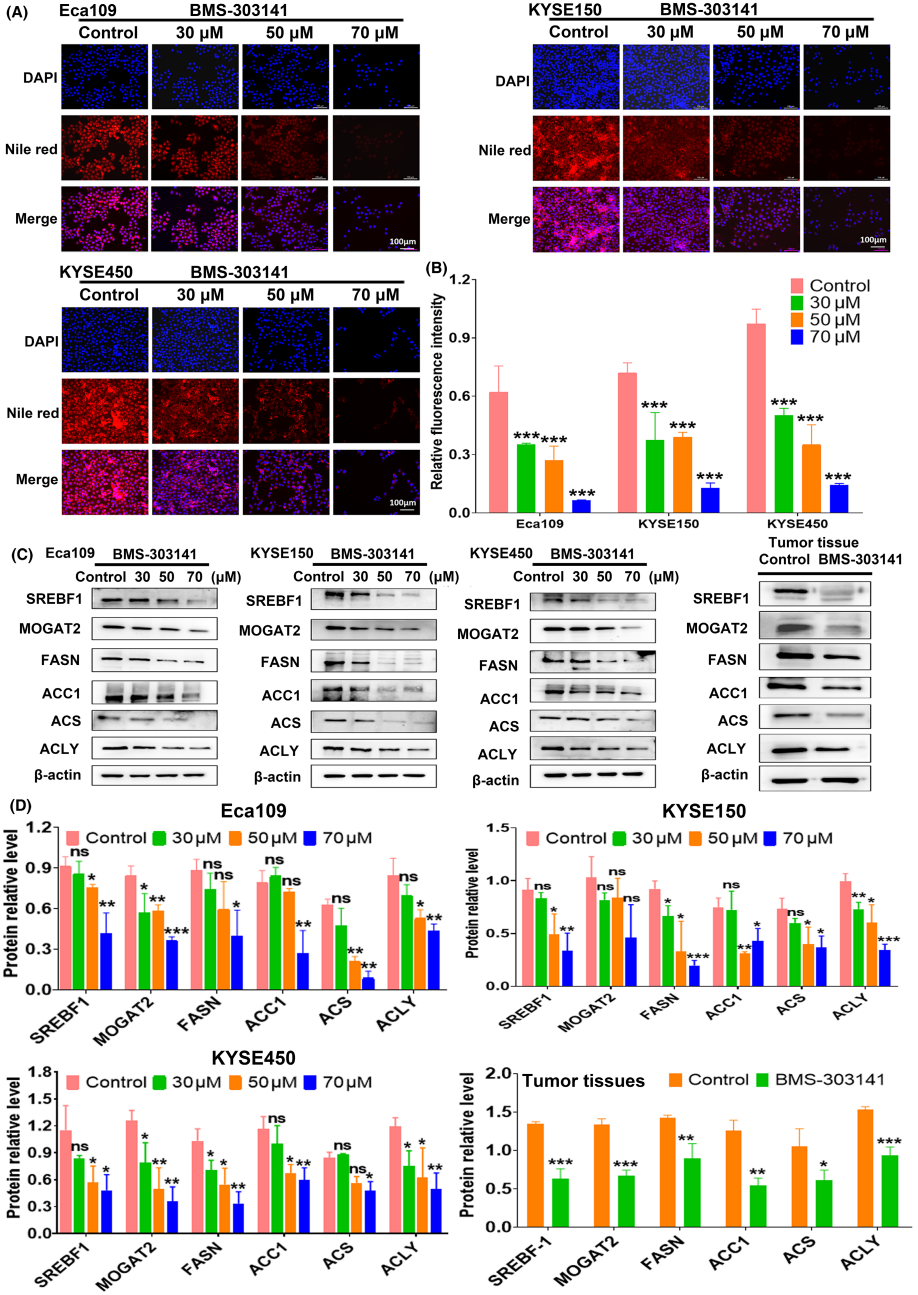
ATP citrate lyase (ACLY) inhibitor suppresses lipogenesis in oesophageal squamous cell carcinoma (ESCC) in vitro and in vivo. (A) Nile red fluorescence staining analysis of lipid content in Eca109, KYSE150 and KYSE450 cells treated with different concentrations of BMS‐303141; (B) Statistical assay for relative lipid content after treatment with different concentrations of BMS‐303141; (C) Western blot was used to detect the effect of BMS‐303141 on the expression of lipid synthesis‐related proteins in ESCC in vitro and in vivo; (D) Relative levels of lipid synthesis‐related proteins in ESCC in vitro and in vivo; *p < 0.05, **p < 0.01 and ***p < 0.001 indicate statistical significance, ns, no significance.

We further explored the effects of ACLY inhibitor on lipid synthesis in ESCC cells using Nile red staining. The results showed that the relative content of lipids in ESCC cells was markedly reduced after treatment with ACLY inhibitor BMS‐303141 (Figure 5A,B). In addition, we further investigated the effects of BMS‐303141 on the expressions of lipid synthesis‐related proteins including SREBF1, MOGAT2, FASN, ACC1, ACS and ACLY in ESCC cells and xenografted tumour tissues by Western blot. The results showed that with the increase of the concentration of BMS‐303141, the expression levels of these lipid synthesis‐related proteins exhibited a gradually declining trend in ESCC cells and tumour tissues (Figure 5C,D). These results suggest that ACLY inhibitor significantly suppresses lipogenesis in ESCC in vitro and in vivo.

### 3.6 ACLY overexpression extremely promotes lipid synthesis in ESCC cells in vitro and in vivo

**FIGURE 6:**
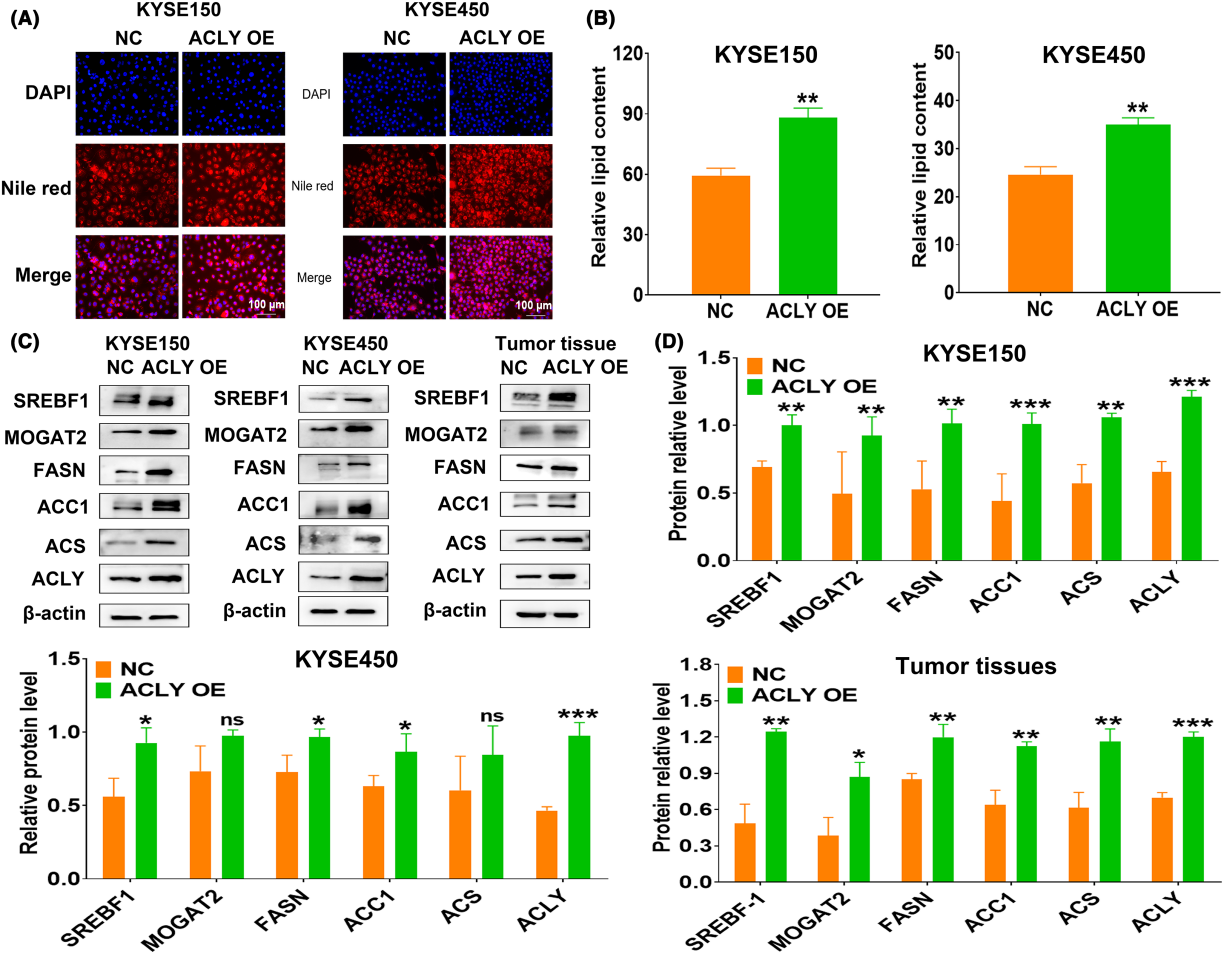
ATP citrate lyase (ACLY) overexpression promotes lipid synthesis in oesophageal squamous cell carcinoma (ESCC) in vitro and in vivo. (A) Nile red fluorescence staining assay for lipid content of ESCC cells in NC and ACLY OE groups; (B) Statistical analysis of lipid content of KYSE150 and KYSE450 cells in NC and ACLY OE groups; (C) Western blot detection for the expressions of lipid synthesis‐related proteins in ESCC cells and tumour tissues; (D) Statistical analysis of the relative levels of lipid synthesis‐related proteins in ESCC cells and tumour tissues; *p < 0.05, **p < 0.01 and ***p < 0.001 indicate statistical significance, ns, no significance.

To investigate whether ACLY overexpression can promote lipid synthesis, Nile red fluorescence staining was also used to detect the changes in lipid content in ESCC cells after ACLY overexpression. The results showed that the relative intensity of red fluorescence in the ACLY OE group was significantly higher than that in the NC group (Figure 6A,B). Stepwise investigation revealed that ACLY overexpression significantly promoted the expression of lipid synthesis‐related proteins in ESCC cells and tumour tissues (Figure 6C,D). These results suggest that ACLY overexpression significantly promotes lipogenesis in ESCC in vitro and in vivo.

### 3.7 ACLY is acetylated and interacts with SIRT2 in ESCC cells

**FIGURE 7:**
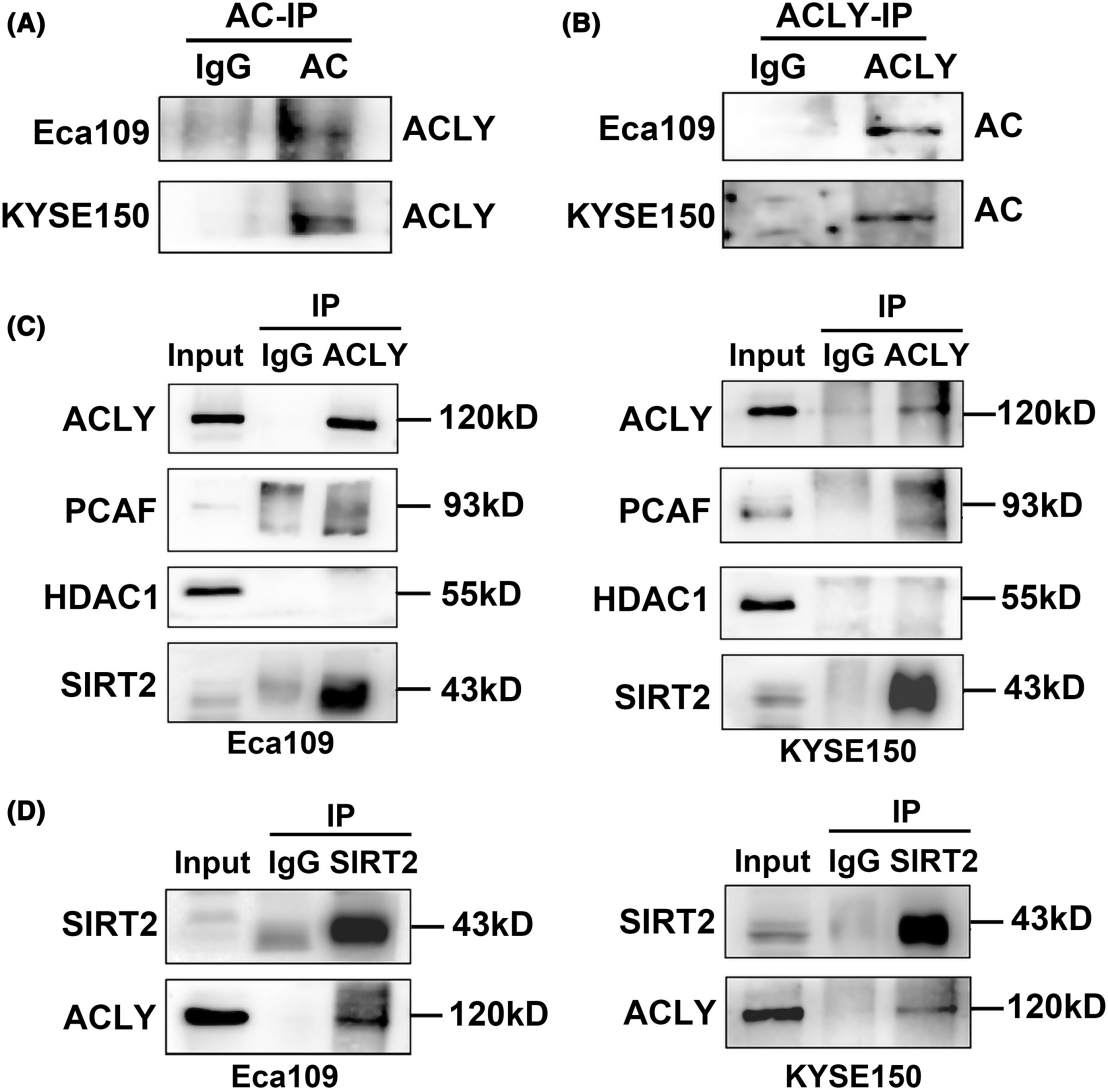
ATP citrate lyase (ACLY) is acetylated and interacts with SIRT2 in oesophageal squamous cell carcinoma (ESCC) cells. (A) ACLY acetylation was detected by Co‐IP using pan‐acetylated antibody (AC) in Eca109 and KYSE150 cells; (B) ACLY acetylation was detected by Co‐IP using ACLY antibody in Eca109 and KYSE150 cells; (C) Co‐IP was used to detect the interaction of ACLY with PCAF, HDAC1 and SIRT2 in Eca109 and KYSE150 cells; (D) Co‐IP was used to detect the interaction between SIRT2 and ACLY using SIRT2 antibody in Eca109 and KYSE150 cells.

Previous reports have shown that ACLY has multiple different types of protein modifications, among which acetylation plays a key role in maintaining protein stability.^22^, ^32^ To explore whether ACLY is acetylated in ESCC cells, Co‐IP was used to detect the acetylation of ACLY in Eca109 and KYSE150 cells by pan‐acetylated antibody (AC) as Co‐IP antibody for immunoprecipitation, and then Western blot was used to detect the expression of ACLY in protein complex. The results showed that ACLY in Eca109 and KYSE150 cells were modified by acetylation (Figure 7A). Similarly, we further confirmed the acetylation of ACLY in Eca109 and KYSE150 cells by immunoprecipitation using ACLY as Co‐IP antibody (Figure 7B). To further explore the enzymes involved in ACLY acetylation modification in ESCC cells, ACLY antibody was used as Co‐IP antibody for immunoprecipitation, and the expressions of acetyltransferases PCAF and deacetylases (HDAC1 and SIRT2) in the complex were detected by Western blot. The results showed that ACLY exhibited significant interaction with PCAF and SIRT2, but not HDAC1 in Eca109 and KYSE150 cells (Figure 7C). Because SIRT2 was significantly enriched in the protein complex, SIRT2 antibody as Co‐IP antibody was utilized for immunoprecipitation for reverse verification. Our results further confirmed the interaction between ACLY and SIRT2 in Eca109 and KYSE150 cells (Figure 7D). These findings suggest that ACLY is acetylated and interacts with SIRT2 in ESCC cells.

### 3.8 SIRT2 mediates deacetylation of ACLY in ESCC cells

**FIGURE 8:**
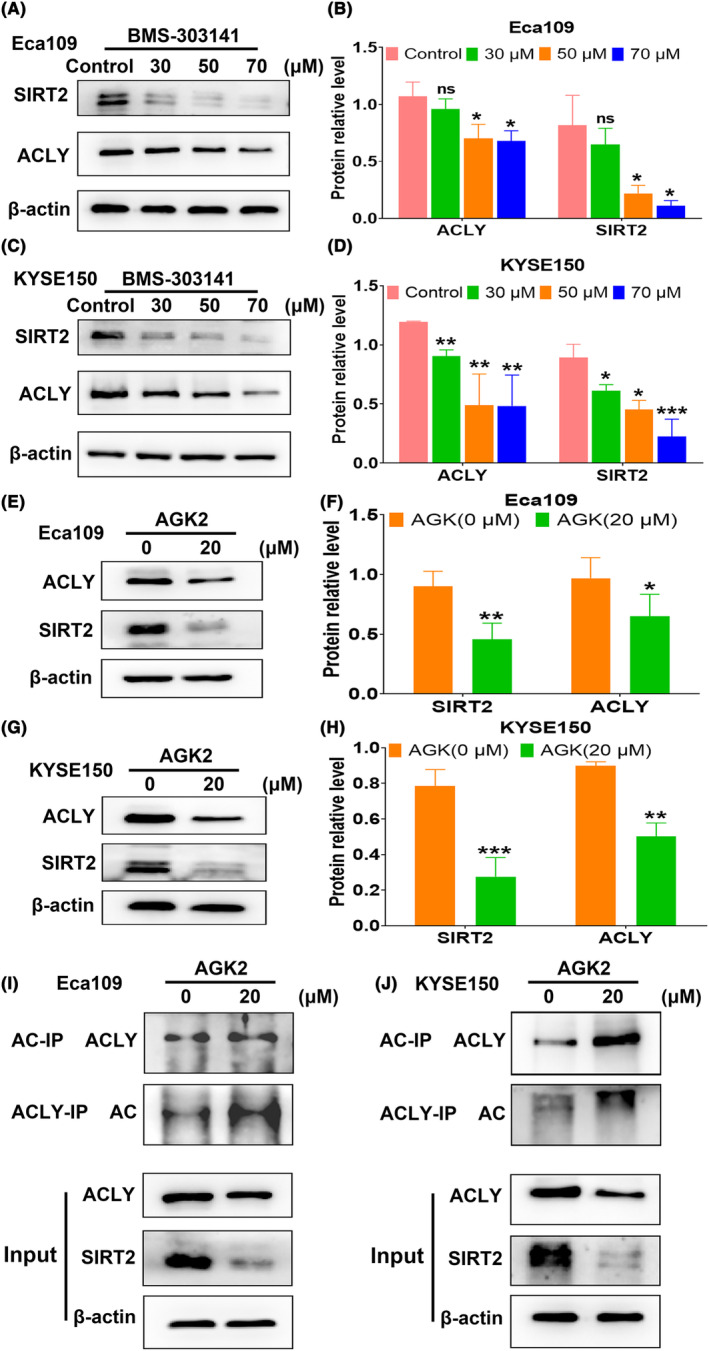
SIRT2 mediates deacetylation of ATP citrate lyase (ACLY) in oesophageal squamous cell carcinoma (ESCC) cells. (A) Western blot detection for the protein expressions of SIRT2 and ACLY in Eca109 cells after treatment with different concentrations of BMS‐303141; (B) Statistical analysis of relative levels of SIRT2 and ACLY in Eca109 cells after treatment with different concentrations of BMS‐303141; (C) Western blot assay for the protein expression of SIRT2 and ACLY in KYSE150 cells after treatment with different concentrations of BMS‐303141; (D) Statistical analysis of relative levels of SIRT2 and ACLY in KYSE150 cells after treatment with different concentrations of BMS‐303141; (E) Western blot assay for the protein expressions of SIRT2 and ACLY in Eca109 cells after SIRT2 inhibitor AGK2 treatment; (F) Statistical analysis of relative levels of SIRT2 and ACLY in Eca109 cells after AGK2 treatment; (G) Western blot detection for the protein expression of SIRT2 and ACLY in KYSE150 cells after AGK2 treatment; (H) Statistical analysis of relative levels of SIRT2 and ACLY in KYSE150 cells after AGK2 treatment; (I) AGK2 enhanced the level of ACLY acetylation in Eca109 cells by Co‐IP experiment; (J) AGK2 enhanced the level of ACLY acetylation in KYSE150 cells by Co‐IP experiment; *p < 0.05, **p < 0.01 and ***p < 0.001 indicate statistical significance, ns, no significance.

To further explore the regulatory relationship between ACLY and SIRT2, Western blot was used to detect the effect of ACLY inhibitor on SIRT2 expression in ESCC cells. We found that BMS‐303141 evidently inhibited the expression of ACLY and SIRT2 in ESCC cells in a dose‐dependent manner (Figure 8A–D). Moreover, SIRT2 inhibitor AGK2 significantly down‐regulated the expressions of SIRT2 and ACLY proteins in ESCC cells (Figure 8E–H). Subsequently, To further investigate whether SIRT2 affected the level of ACLY acetylation in ESCC cells, Eca109 and KYSE150 cells were treated with SIRT2 inhibitor AGK2 and the acetylation level of ACLY was detected. We found that the acetylation level of ACLY was increased after AGK2 treatment, but the protein expression level of ACLY was down‐regulated (Figure 8I,J). These results suggest that SIRT2 mediates ACLY deacetylation in ESCC cells.

### 3.9 ACLY overexpression partially reversed the inhibitory effect of AGK2 on proliferation and migration of ESCC cells

**FIGURE 9:**
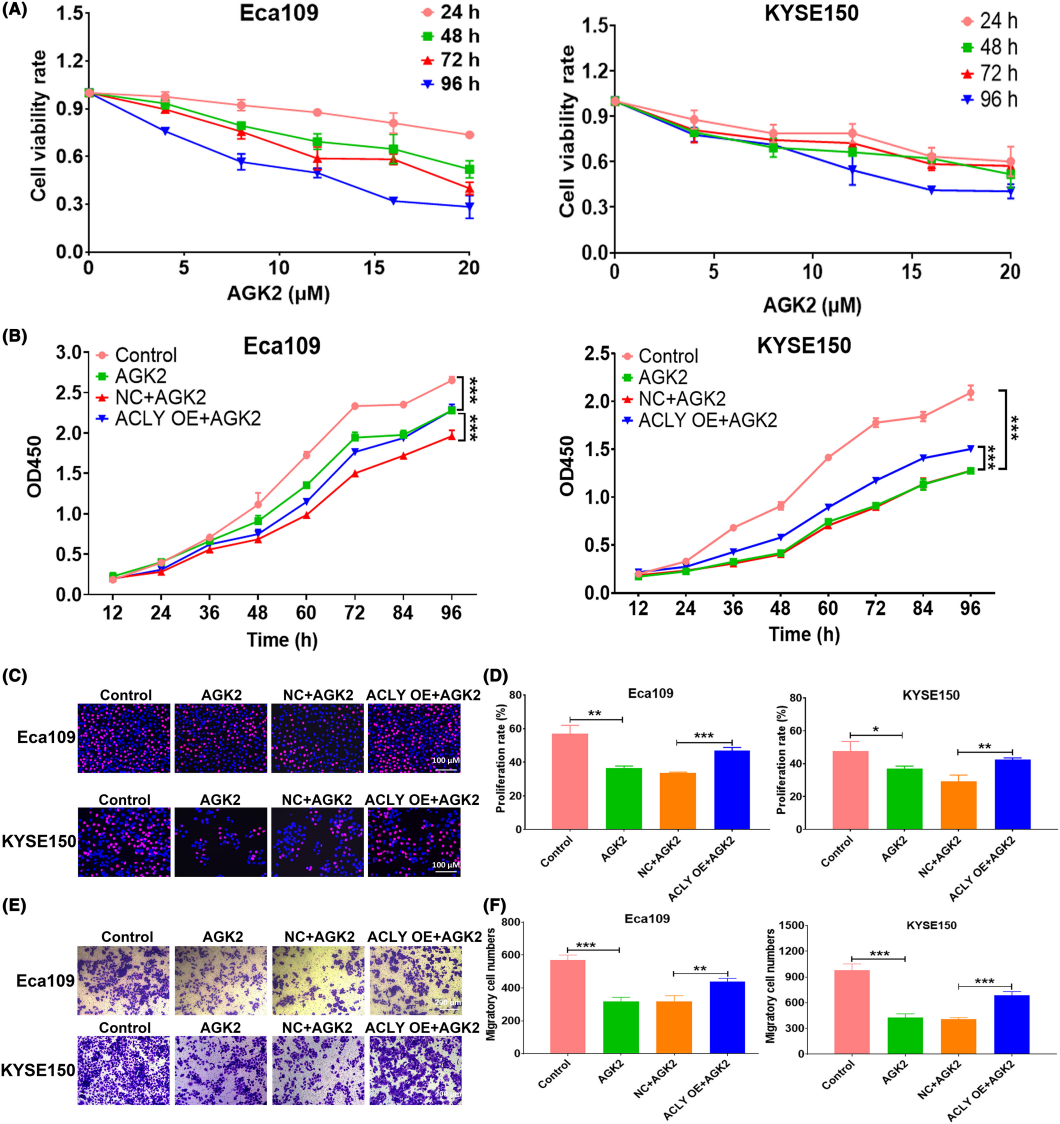
ATP citrate lyase (ACLY) overexpression partially reverses the inhibitory effect of AGK2 on proliferation and migration of oesophageal squamous cell carcinoma (ESCC) cells. (A) CCK‐8 was used to detect the effects of different concentrations of AGK2 on the survival rate of Eca109 and KYSE150 cells; (B) CCK‐8 was used to detect cell proliferation ability in Eca109 and KYSE150 cells with different treatments (Control group, AGK2 group, NC + AGK2 group and ACLY OE + AGK2 group); (C) EdU staining was used to detect the proliferation rate of Eca109 and KYSE150 cells in Control group, AGK2 group, NC + AGK2 group and ACLY OE + AGK2 group; (D) Statistical assay for EdU‐positive cell numbers in Control group, AGK2 group, NC + AGK2 group and ACLY OE + AGK2 group; (E) Transwell experiment was used to detect the migratory ability of Eca109 and KYSE150 cells in Control group, AGK2 group, NC + AGK2 group and ACLY OE + AGK2 group; (F) Statistical assay for migratory cell numbers in Control group, AGK2 group, NC + AGK2 group and ACLY OE + AGK2 group; *p < 0.05, **p < 0.01 and ***p < 0.001 indicate statistical significance.

To further explore the role of SIRT2 inhibitor AGK2 in the proliferation of ESCC cells, CCK‐8 was used to detect the effects of different concentrations of AGK2 on the cell viability of ESCC cells. The results showed that AGK2 significantly inhibited the cell viability of Eca109 and KYSE150 in a time‐ and dose‐dependent manner (Figure 9A). Based on the antitumor effect of SIRT2 inhibitor AGK2 in ESCC, this study further investigated whether ACLY overexpression could reverse the antitumor effect of AGK2. CCK‐8 and EdU were used to detect cell proliferation in Control group, AGK2 group, NC + AGK2 group and ACLY OE + AGK2 group. The results showed that AGK2 could significantly inhibit the proliferation of Eca109 and KYSE150 cells, while ACLY overexpression could partially reverse AGK2‐mediated proliferation inhibition of ESCC cells (Figure 9B–D). In addition, transwell assay showed that AGK2 significantly inhibited ESCC cell migration, and ACLY overexpression partially reversed AGK2‐mediated inhibition of ESCC cell migration (Figure 9E,F). These results indicate that AGK2 significantly inhibits the proliferation and migration of ESCC cells, whereas the overexpression of ACLY could partially reverse the inhibitory effect above.

## 4 DISCUSSION

Increased lipid uptake and storage in malignant tumours are commonly used to meet the needs of the growth of tumour cells. Due to the rapid proliferation of tumours, many tumour cells need to rely on the synthesis of de novo fatty acids, which are synthesized into new cell membranes for proliferating cells, and the excess fatty acids are also involved in the regulation of tumour cell signal transduction.^33^ ACLY, as the “initiation” enzyme of de novo fatty acid synthesis, plays an extremely key role in tumour fat synthesis and participates in tumour progression and metastasis. Studies have shown that ACLY is abnormally expressed in breast cancer, prostate cancer, bladder cancer, lung cancer, gastric cancer, liver cancer and colon cancer.^27^, ^34^, ^35^, ^36^, ^37^, ^38^, ^39^ In addition, low ACLY expression predicts a good prognosis in patients with acute myeloid leukaemia.^40^ These studies suggested that ACLY was closely related to tumour development and progression. To further explore the role of ACLY in ESCC, we analysed the expression of ACLY in oesophageal cancer by bioinformatics and molecular biological methods. We found that ACLY was found to be highly expressed in a variety of cancers. Moreover, StarBase V3.0, UALCAN and GEPIA databases from different TCGA sources all showed high expression of ACLY in ESCA. Further qRT‐PCR analysis of 31 ESCC samples showed that 16 cases exhibited high expression of ACLY, which was also validated in ESCC cells. These findings suggest that ACLY may participate in the development and progression of ESCC, however, whether ACLY may be a novel prognostic biomarker for ESCC patients remains under investigation.

Increasing evidence has shown that high ACLY expression is tightly involved in the regulation of multiple different biological processes, such as cell proliferation, migration, lipogenesis and drug resistance, and is associated with poor prognosis.^28^, ^29^, ^41^, ^42^, ^43^, ^44^ To explore the functions of ACLY in ESCC, gain‐of‐function and loss‐of‐function experiments revealed that ACLY inhibitor BMS‐303141 significantly inhibited the proliferation, migration and invasion of ESCC, whereas ACLY overexpression significantly promoted the proliferation, migration and invasion of ESCC cells. Most importantly, we also confirmed that the ACLY inhibitor BMS‐303141 significantly inhibited the growth of xenografts in nude mice through in vivo animal experiments, but ACLY overexpression promoted the growth of xenografts in nude mice. These data suggest that targeting ACLY may be a promising therapeutic approach for ESCC patients. Lipids are not only the main components of cell membranes, but are also used for energy storage and metabolism, and play an important role in signal regulation activities of many tumour cells.^33^ Regulation of lipid metabolism, such as lipid uptake, synthesis and breakdown, is essential for maintaining cellular homeostasis, and tumour cells maintain their rapid proliferation, survival, migration, invasion and metastasis by lipid metabolism.^15^ ACLY, ACC1, FASN, HMGCR and other enzymes play an important role in lipid metabolism. Sato et al.^45^ confirmed that ACLY was the target gene of SREBF1, and SREBF1 transcriptional regulation of ACLY enzyme activity generated cytosolic acetyl‐CoA required for cholesterol and fatty acid synthesis. To further investigate the role of ACLY in lipid metabolism in ESCC, we firstly observed lipid biosynthesis by Nile red staining, the results showed that ACLY inhibitor significantly suppressed lipid production in ESCC cells, but ACLY overexpression had the opposite effect. Further, we detected the expression of lipid‐related proteins ACLY, ACS, ACC1, FASN, MOGAT2 and SREBF1 in vitro and in vivo, and found that ACLY inhibitor significantly inhibited the expressions of these lipid‐related proteins, whereas ACLY overexpression significantly promoted the expression of lipid‐related proteins. These results indicate that ACLY plays an important role in the lipid metabolism of ESCC, and it affects the growth and invasion ability of ESCC by regulating the expression of lipid‐related proteins, but the exact molecular mechanism needs to be further studied.

ACLY is a homotetramer with 1101 amino acid residues in each polypeptide and has multiple acetylation, ubiquitination and phosphorylation modification sites.^46^ Studies have shown that HRD1 interacts with ACLY in non‐alcoholic fatty liver disease, thereby promoting ACLY ubiquitination leading to its degradation.^47^ The interaction between USP30 and ACLY leads to the deubiquitination of ACLY, resulting in the increased stability of ACLY, and thereby promoting adipogenesis, inflammation and tumorigenesis in hepatocellular carcinoma.^41^ Acetylation of ACLY at Lys‐540, Lys‐546 and Lys‐554 (ACLY‐3 K) can antagonize ubiquitination of ACLY, leading to increased stability of ACLY protein, and further promoting lipid synthesis and cell proliferation of lung cancer cells.^32^ To further explore whether ESCC cells have ACLY acetylation modification, Co‐IP was used to detect the acetylation modification of ACLY, and we found that ACLY had acetylation modification. Acetylation and deacetylation of lysine residues are reversibly regulated by acetyltransferase and deacetylase, which are implicated in tumour progression and metastasis.^48^ To further unearth the enzymes related to the regulation of ACLY acetylation level, acetyltransferase (PCAF) and deacetylase (HDAC1 and SIRT2) were selected for further study, we found that ACLY had significant interaction with PCAF and SIRT2 in ESCC cells, but not HDAC1. However, because SIRT2 was significantly enriched in the protein complex, we used SIRT2 as Co‐IP antibody for reverse verification by immunoprecipitation. The interaction between ACLY and SIRT2 in ESCC cells was further confirmed. Although SIRT2 may be an important deacetylase for regulating the level of ACLY acetylation in ESCC cells, current reports about other sirtuin protein families, such as SIRT6, also regulate the level of ACLY acetylation.^49^ However, whether SIRT6 or other sirtuin families such as SIRT1, SIRT3, etc. can regulate the ACLY acetylation remains to be elucidated. Overall, our current findings suggest that SIRT2 may play a role in manipulating the level of ACLY acetylation in ESCC cells.

Currently, acetylation of ACLY at Lys‐540, Lys‐546 and Lys‐554 makes ACLY more stable by antagonizing ACLY ubiquitination, and ACLY‐3K acetylation can also promote the stability of ACLY protein in mouse liver cell line AML12, while SIRT2 promotes the deacetylation of ACLY‐3K, resulting in the reduction of ACLY stability.^50^ In the present study, BMS‐303141 inhibited the expression of SIRT2, and AGK2 also down‐regulated the expression of ACLY. In addition, the acetylation level of ACLY in ESCC cells was enhanced after SIRT2 inhibition, but the expression of ACLY was down‐regulated. Further investigation revealed that AGK2 significantly inhibited the proliferation and migration of ESCC cells, whereas ACLY overexpression partially reversed the inhibitory effect. The possible reasons for our current data were due to the fact that the acetylation site of ACLY has not been studied in our current study, and the same modification at different sites could have completely different effects on protein function.^51^ These results suggest that SIRT2 may mediate the deacetylation of ACLY in ESCC cells and thus participate in the progression of ESCC.

## 5 CONCLUSION

**FIGURE 10:**
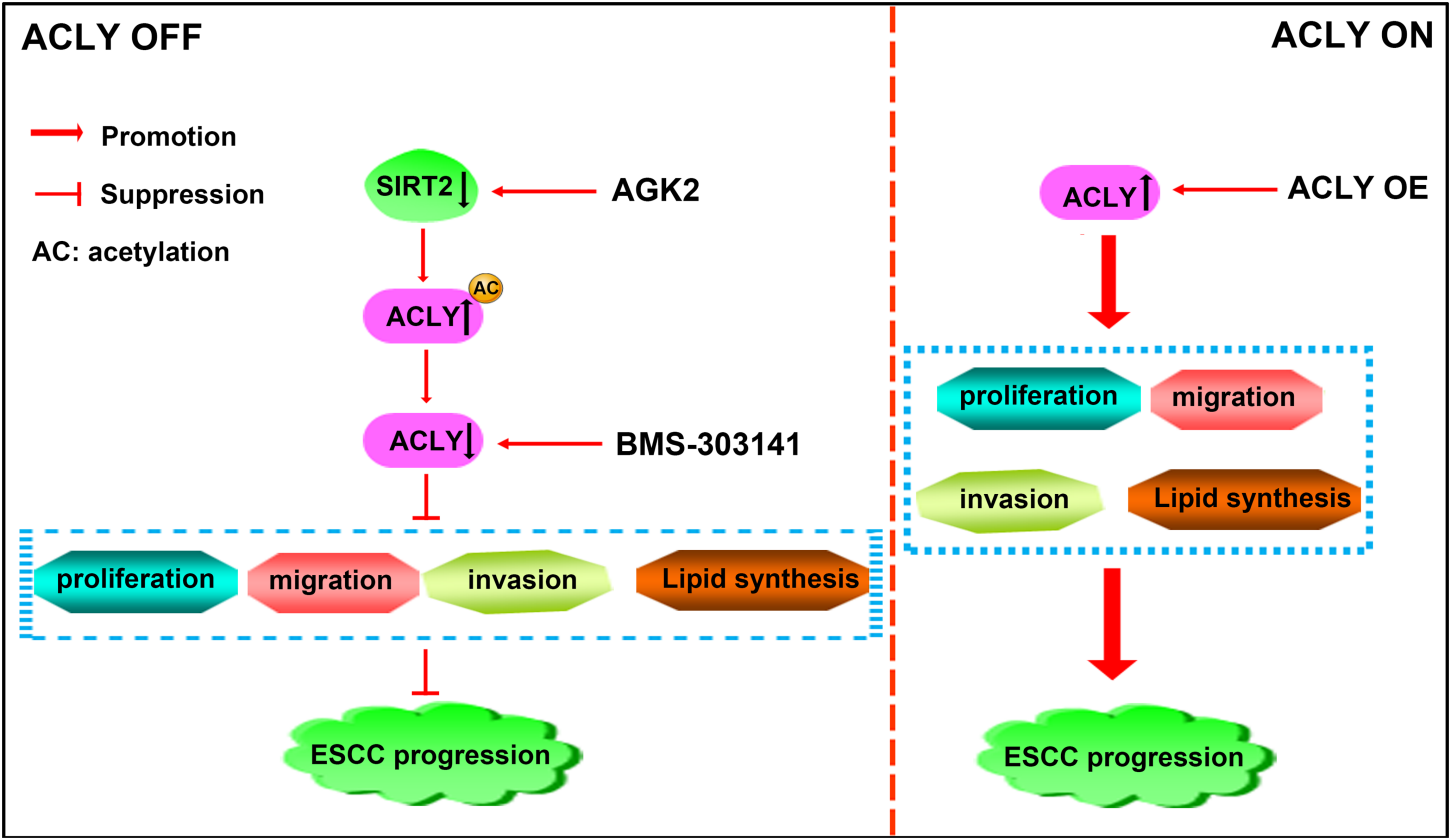
Schematic representation of the mechanism of action of ATP citrate lyase (ACLY) in oesophageal squamous cell carcinoma (ESCC). SIRT2 inhibitor AGK2 resulted in the upregulation of the level of ACLY acetylation and the downregulation of ACLY protein level, and BMS‐303141 evoked the downregulation of ACLY protein, which suppressed cell proliferation, migration, invasion, lipid synthesis and the growth of xenografted tumour in ESCC cells, further resulting in the suppression of ESCC progression. Furthermore, ACLY overexpression promoted cell proliferation, migration, invasion, lipid synthesis and the growth of xenografted tumour in ESCC cells, further promoting ESCC progression.

In conclusion, our preliminary results suggest that ACLY inhibitor BMS‐303141 suppresses cell proliferation, migration, invasion, lipid synthesis and the growth of xenografted tumour in ESCC cells, whereas ACLY overexpression displays the opposite effects (Figure 10). Mechanistically, ACLY harbours acetylation modification, and SIRT2 interacts with ACLY in ESCC cells. SIRT2 inhibitor AGK2 evokes the upregulation of the level of ACLY acetylation and the downregulation of ACLY protein level, further resulting in the suppression of ESCC progression (Figure 10). Our data presented herein suggest that targeting SIRT2/ACLY signalling axis may have important clinical value and provide a new strategy for targeted therapy of ESCC patients.

## AUTHOR CONTRIBUTIONS

**Xueying Zhang:** Data curation (equal); investigation (equal); methodology (equal); validation (equal). **Yue Xu:** Data curation (equal); investigation (equal); methodology (equal); validation (equal). **Shenglei Li:** Data curation (equal); funding acquisition (equal); investigation (equal); methodology (equal); validation (equal). **Yue Qin:** Formal analysis (equal); software (equal); validation (supporting). **Guangzhao Zhu:** Formal analysis (equal); software (equal); validation (supporting). **Qing Zhang:** Funding acquisition (equal); methodology (supporting); validation (supporting). **Yanting Zhang:** Funding acquisition (equal); methodology (supporting); validation (supporting). **Fangxia Guan:** Conceptualization (equal); funding acquisition (equal); project administration (equal); supervision (equal); writing – review and editing (lead). **Tianli Fan:** Conceptualization (equal); funding acquisition (equal); project administration (equal); supervision (equal); writing – original draft (equal). **Hongtao Liu:** Conceptualization (equal); funding acquisition (equal); project administration (equal); supervision (equal); writing – original draft (equal).

## FUNDING INFORMATION

This study was supported by the National Natural Science Foundation of China (82073084 and 81272691), a Key project of Henan Provincial Science and Technology Research and Development Joint Fund (225200810011), Joint Construction Project of Henan Medical Science and Technology Research Program (LHGJ20210701), the Key Discipline Construction Project for Prevention and Treatment of Oesophageal Cancer in Zhengzhou University (XKZDJC202001), the Central Plains Thousand People Plan of Henan Province (204200510013), the Discipline Innovation and Wisdom Introduction Plan of Higher Education in Henan Province (CXJD2021002), the Natural Science Foundation of Henan Province (202300410404).

## CONFLICT OF INTEREST STATEMENT

The authors declare no conflicts of interest.

## Data Availability

The data sets used and/or analysed during the current study are available from the corresponding author upon reasonable request.
